# Introduction of a Fit for Purpose Induction Booklet to Improve Junior Doctor’s/Trainee’s Experience of Local Induction Programme

**DOI:** 10.1192/bjo.2025.10330

**Published:** 2025-06-20

**Authors:** Nermeen Ahmed, Ismail Memon

**Affiliations:** Swansea Bay University Health Board, Swansea, United Kingdom

## Abstract

**Aims:** The term ‘Black Wednesday’ has been used to describe the August national changeover day for nearly 50,000 junior doctors in the NHS, a day when a new cohort of inexperienced doctors start work. Junior trainees have reported a 7–14% reduced satisfaction compared with higher specialist trainees.

Doctors need to be supported in the workplace to provide safe and high-quality patient care. Induction as a minimum should introduce trainees to organisational policies, procedures and arrangements for clinical governance, orientation, and support. Although there is an existing induction system, having a written structured manual will assist the trainees to get through this process more easily. This project aimed to develop an Induction handbook containing all necessary information and links for trainees in Psychiatry at SBUHB to improve trainees’ overall satisfaction of the induction programme.

**Methods:** Data was collected through baseline pre-QI questionnaire which was analysed by a Pareto chart. Following that the induction handbook was circulated to the trainees and a post-QI questionnaire was completed and final data was analysed and compared against the pre-QI results

The project had one PDSA cycle. During which we approached different mental health services directorates within SBUHB as well as community and inpatient consultants for their input. Policies search was carried out on the health board intranet and SharePoint drive.

**Results:** The post-QI questionnaire receiving 15/18 respondents showed: 60% respondents rated very satisfied, 26.7% satisfied while 13.3% rated neutral; 60% respondents strongly agreed and 26.7% agreed that the handbook will help in safe delivery of patient care while 13.3% rated neutral.

**Conclusion:** The Post-QI survey has successfully confirmed that the updated and improved induction handbook has helped to improve the trainee’s satisfaction and overall experience by having a more comprehensive induction; as it has all the necessary information required to guide them through different systems and processes. Ultimately, finding it very helpful to get the required training to deliver the safe and effective patient care needed at their new placements.

